# Genetic Targeting of GRP78 in the VMH Improves Obesity Independently of Food Intake

**DOI:** 10.3390/genes9070357

**Published:** 2018-07-17

**Authors:** Laura Liñares-Pose, Eva Rial-Pensado, Ánxela Estévez-Salguero, Edward Milbank, Ismael González-García, Claudia Rodríguez, Patricia Seoane-Collazo, Noelia Martinez-Sánchez, Rubén Nogueiras, Dolores Prieto, Carlos Diéguez, Cristina Contreras, Miguel López

**Affiliations:** 1 Department of Physiology, CIMUS, University of Santiago de Compostela-Instituto de Investigación Sanitaria de Santiago de Compostela, 15782 Santiago de Compostela, Spain; laura.linares@usc.es (L.L.-P.); eva.pensado@usc.es (E.R.-P.); anxela.estevez@usc.es (Á.E.-S.); ed.milbank@usc.es (E.M.); ismael.gonzalez@usc.es (I.G.-G.); patricia.seoane@usc.es (P.S.-C.); noelia.martinez@usc.es (N.M.-S.); ruben.nogueiras@usc.es (R.N.); carlos.dieguez@usc.es (C.D.); 2 CIBER Fisiopatología de la Obesidad y Nutrición (CIBERobn), 15706 Santiago de Compostela, Spain; 3 Department of Physiology, School of Pharmacy, Universidad Complutense de Madrid, 28040 Madrid, Spain; claudrod@ucm.es (C.R.); dprieto@farm.ucm.es (D.P.)

**Keywords:** brown adipose tissue, browning, ER stress, GRP78, hypothalamus, thermogenesis, white adipose tissue

## Abstract

Recent data have demonstrated that the hypothalamic GRP78/BiP (glucose regulated protein 78 kDa/binding immunoglobulin protein) modulates brown adipose tissue (BAT) thermogenesis by acting downstream on AMP-activated protein kinase (AMPK). Herein, we aimed to investigate whether genetic over-expression of GRP78 in the ventromedial nucleus of the hypothalamus (VMH: a key site regulating thermogenesis) could ameliorate very high fat diet (vHFD)-induced obesity. Our data showed that stereotaxic treatment with adenoviruses harboring GRP78 in the VMH reduced hypothalamic endoplasmic reticulum ER stress and reversed vHFD-induced obesity. Herein, we also demonstrated that this body weight decrease was more likely associated with an increased BAT thermogenesis and browning of white adipose tissue (WAT) than to anorexia. Overall, these results indicate that the modulation of GRP78 in the VMH may be a target against obesity.

## 1. Introduction

Energy balance can be modulated by peripheral signals acting on the central nervous system (CNS), notably on the hypothalamus [1,2,3]. In the last decade, there has been a growing interest in mechanisms activating the thermogenic process, particularly on the central control of the brown adipose tissue (BAT) activity [3,4,5,6,7]. Even more recently, browning—a process described as the trans-differentiation of white into beige/brite adipocytes in the white adipose tissue (WAT)—has been recognized to be a therapeutic target to reduce body weight through the increase of energy expenditure [8,9,10,11].

Increasing evidence has revealed a strong interaction between hypothalamic endoplasmic reticulum (ER) stress and obesity occurrence. ER stress is closely associated with obesity-associated insulin resistance in peripheral tissues, such as pancreas and liver [12,13,14]. Hypothalamic ER stress occurs under conditions of nutritional excess, such as obesity and type 2 diabetes, which are associated with leptin and insulin resistance [15,16,17,18,19]. Since improvements of hypothalamic ER stress, either through pharmacological or genetic increases of protein folding, have been associated with a body weight decrease and to an enhancement of leptin and insulin sensitivity, targeting this mechanism opened new potential therapeutic avenues [15,16,17,20,21]. In the same line of findings, recent data have also demonstrated that central ceramide-induced lipotoxicity increases ER stress contributing to weight gain, glucose intolerance, decreased sympathetic tone, and impaired BAT thermogenesis [17,22,23]. Interestingly, the central effects of ceramides can be reversed by decreasing ER stress, particularly in the ventromedial nucleus of the hypothalamus (VMH) [17], a key site implicated in the modulation of BAT thermogenesis [3,6,7,24].

The general objective of this study was to investigate whether a genetic over-expression of glucose regulated protein 78 kDa (GRP78) in the VMH could improve very high fat diet (vHFD: extreme fat content, providing 60% of energy as fat)-induced obesity associated complications. Our data demonstrated that a stereotaxic injection of adenoviruses harboring GRP78 in the VMH could reduce hypothalamic ER stress and reverse obesity in rats chronically fed with a vHFD. Overall, these results confirm that the modulation of GRP78 in the VMH is an effective target against obesity. 

## 2. Materials and Methods

### 2.1. Animals

For the further described experiments, 50 g male Sprague-Dawley rats (4–5 weeks old, Animalario General USC, Santiago de Compostela, Spain) were used. All animals were housed in the same room on a 12:12-h light dark period at temperature and humidity-controlled conditions, and they were allowed free access to chow and tap water. Sprague-Dawley rats were separated in two different feeding groups: (i) the first group was submitted to a standard laboratory diet (STD, SAFE A04: 3.1% fat, 59.9% carbohydrates, 16.1% proteins, 2.791 kcal/g; Scientific Animal Food & Engineering; Nantes, France), and (ii) the second one to a very high fat diet (vHFD, D12492: 60% fat, 20% carbohydrate, 20% protein, 5.21 kcal/g; Research Diets, Inc.; New Brunswick, NJ, USA). The animals were housed collectively in groups of 4/cage for a period of 3 months under these dietary conditions until the beginning of the experiments. In all the experimental settings, the animals were individually housed and their respective food consumption (STD or vHFD) and body weight were daily measured. The experiments were performed in agreement with the International Law on Animal Experimentation and were approved by the USC Ethical Committee (Project License 15010/14/006)*.*

### 2.2. Stereotaxic Microinjection of Adenoviruses

Rats were deeply anesthetized (intraperitoneal ketamine-xylazine injection) and the head was located in a stereotaxic frame (David Kopf Instruments; Tujunga, CA, USA). The VMH was bilaterally targeted using a 25-gauge needle (Hamilton; Reno, NV, USA). The cannula was implanted in the VMH following already reported stereotaxic coordinates: 2.4/3.2 mm posterior to the bregma, ±0.6 mm lateral to midline and 10.1 mm ventral [4,5,17,23,24,25,26]. Adenoviral vectors (Viraquest; North Liberty, IA, USA) encoding for green fluorescence protein (GFP, used as control) or GRP78 and GFP (both at 10^12^ pts/mL) were injected at a flow of 200 nL/min during 5 min (1 µL/injection site) as previously described [4,5,17,23,24,25,26]. Animals were observed for 7 days; 8–11 rats per group were used. 

### 2.3. Temperature Measurements

Body temperature was recorded two times at the end of the experiments with a rectal probe coupled to a digital thermometer (BAT-12: Microprobe-Thermometer; Physitemp, NJ, USA). The surrounding BAT skin temperature was measured using an infrared camera (B335: Compact-Infrared-Thermal-Imaging-Camera; FLIR; West Malling, Kent, UK) and analyzed with the specific associated software (FLIR-Tools-Software; FLIR; West Malling, Kent, UK) [5,17,23,24,25,26,27].

### 2.4. Glucose and Insulin Tolerance Tests

At the end of the treatments, insulin (ITT) and glucose (GTT) tolerance tests were performed. For the ITT, an intraperitoneal injection of 0.75 U/kg insulin (Actrapid, Novonordisk; Bagsvaerd, Denmark) was given and blood glucose levels were recorded at different time points (0, 10, 20, 30, and 60 min), as shown [17,23]. For the GTT, overnight fasted animals were orally administered by gavage with 2 mg/g d-glucose (Sigma; St. Louis, MO, USA) and blood glucose levels were measured at 0, 15, 30, 60, and 90 min, as reported [17,23]. In both ITT and GTT, blood glucose levels were recorded using a glucometer (Accucheck; Roche; Barcelona, Spain).

### 2.5. Sample Processing

Animals were sacrificed by cervical dislocation and decapitation. For each animal, the VMH, liver, the interscapular BAT, the gonadal WAT (gWAT), and the subcutaneous WAT (sWAT, from the inguinal area) were collected for further described analysis (Western blotting, oil red O staining and real-time PCR analysis). The tissues were directly homogenized on ice to maintain the levels of phosphorylation of the proteins. The collected tissues and serum were stored until use at −80 °C. Dissection of the VMH was performed by micropunch procedure under the microscope, as previously described [4,5,17,23,24,25]. For immunohistochemical analysis, the samples originating from gWAT and sWAT were submitted to a 24 h 10% formalin bath followed by an immersion in ethanol 70%.

### 2.6. Western Blotting

Homogenized VMH and BAT were lysed with appropriate buffers, as previously shown [1,4,5,17,23,24,25,26,27]. Protein lysates were subjected to SDS-PAGE, electrotransferred on a PVDF membrane and incubated with the following antibodies: GRP78 (1:1000; ref. 3183), (Cell Signaling, Danvers, MA, USA) ATF6α (activating transcription factor 6 alpha) (1:1000; ref. sc-22799), pPERK (Thr981; phosphorylated PKR-like ER kinase) (1:500; ref. sc-32577), peIF2α (Ser52; phosphorylated eukaryotic initiation factor 2 alpha) (1:2000; ref. sc-101670), CHOP (C/EBP homologous protein) (1:1000; ref. sc-793) (Santa Cruz; Santa Cruz, CA, USA), pIRE1α (Ser724; phosphorylated inositol-requiring enzyme 1 alpha) (1:1000; ref. ab48187), UCP1 (uncoupling protein 1) (1:10,000; ref. ab10983) (Abcam; Cambridge, UK), β-actin (1:5000; ref. A5316), and α-tubulin (1:5000; ref. T5168) (Sigma; St. Louis, MO, USA) as previously described [4,5,17,23,24,25,27,28]. The signals were relatively expressed using α-tubulin (for BAT) or β-actin (for VMH) protein levels. In the gel autoradiographic images, all the bands for each picture come always from the same gel, but they may be spliced for clarity, as indicated in the figure legends.

### 2.7. Real-Time PCR

Real-time PCR analysis (TaqMan^®^; Applied Biosystems; Carlsbad, CA, USA) was performed as previously described [1,4,5,17,25,27,28] using specific primers and probes (Supplemental Table S1). Values were indicated relatively to the Hypoxanthine-guanine phosphoribosyltransferase (HPRT) corresponding levels.

### 2.8. Immunohistochemistry

WAT UCP1 expression was evaluated using anti-UCP1 antibody (1:500; ref. ab10983; Abcam, Cambridge, UK) as previously reported [23,29,30]. Hepatic lipid content was investigated through oil red O staining, as previously shown [17,24,28,31,32,33]. The pictures were obtained using a digital camera Olympus XC50 (Olympus Corporation; Tokyo, Japan) at 20×. Digital liver and WAT obtained images were analyzed with ImageJ Software (National Institutes of Health; Bethesda, MD, USA). GFP fluorescence was observed after perfusion of the animals and detected with a fluorescence microscope Olympus IX51 [4,17,23,24,25,26,27,30]. 

### 2.9. Statistical Analysis

Results are expressed as mean ± SEM. Messenger RNA (mRNA) and protein levels were expressed in percentage of control (STD or GFP) rats. Statistical analysis was achieved by *t*-Student test (when two groups were compared) or ANOVA and post-hoc Bonferroni tests (when more than 2 groups were compared). The differences were considered significant when *p* < 0.05.

## 3. Results

### 3.1. GRP78 in the VMH Decreased ER Stress and Body Weight of vHFD Rats

**Figure 1:**
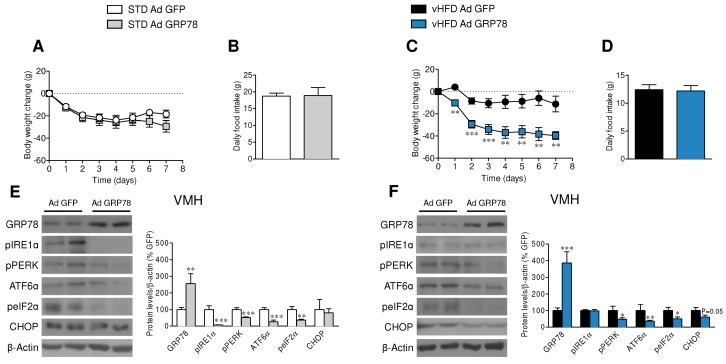
Effect of glucose regulated protein 78 kDa (GRP78) overexpression in the ventromedial nucleus of the hypothalamus (VMH) of very high fat diet (vHFD) rats on energy balance. (**A**,**C**) Body weight change, (**B**,**D**) average daily food intake (*n* = 9–10 animals per group) and (**E**,**F**) representative Western blot autoradiographic images and corresponding VMH protein levels of endoplasmic reticulum (ER) unfolding protein response (UPR) pathway (*n* = 7 animals per group) of standard laboratory diet (STD) or vHFD rats stereotaxically treated with green fluorescence protein (GFP) or GRP78 adenoviruses into the VMH. Statistical significance was determined by *t*-Student test. Error bars represent the SEM. * *p* < 0.05, ** *p* < 0.01 and *** *p* < 0.001 vs. (STD or vHFD) Ad GFP. For the Western blot analysis, representative images for all proteins are shown; in the case of the loading controls a representative gel is displayed, although each protein was corrected by its own internal control (β-actin). In the gel images, all the bands for each picture come always from the same gel, but they may be spliced for clarity.

The injection of GRP78 adenoviruses into the VMH induced feeding-independent weight loss in vHFD rats but not in STD rats (Figure 1A–D). GRP78 adenoviruses infection efficiency in the VMH was assessed by the expression of GFP [4,17,23,24,25,26,27,30], and by increased concentrations of GRP78 (which was higher in the vHFD rats, likely due to the greater basal levels [23]) and reduced ER stress in the VMH, reducing the protein levels of pPERK, pIRE1α, ATF6α, and peIF2α in the VMH of STD rats (Figure 1E) while decreasing pPERK, ATF6α, peIF2α, and CHOP vHFD rats (Figure 1F).

### 3.2. GRP78 in the VMH Stimulated Thermogenesis in the BAT of vHFD Rats

**Figure 2:**
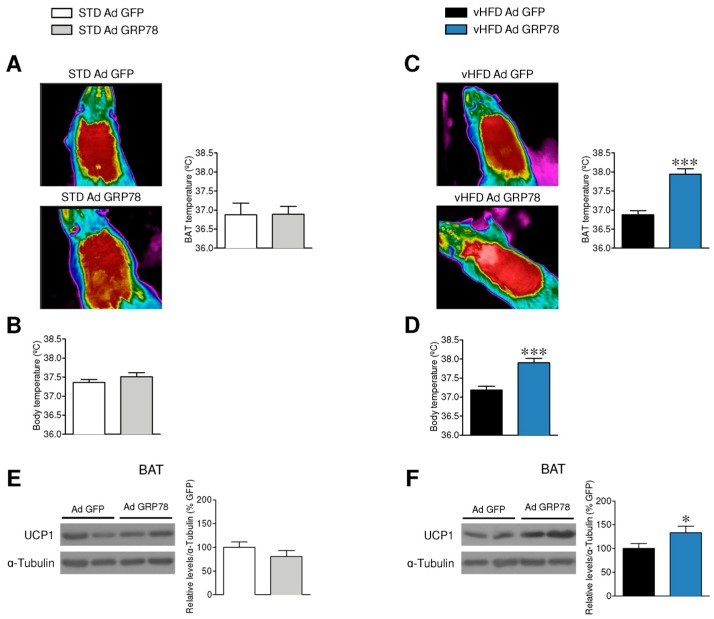
Effect of GRP78 overexpression in the VMH of vHFD rats on brown adipose tissue (BAT) thermogenesis. (**A**,**C**) Representative infrared thermal images (left panel) and temperature of the BAT area (right panels; *n* = 9–10 animals per group), (**B**,**D**) rectal temperature (*n* = 8–9 animals per group) and (**E**,**F**) representative Western blot autoradiographic images and corresponding BAT protein levels of UCP1 (*n* = 7 animals per group) of STD or vHFD rats stereotaxically treated with GFP or GRP78 adenoviruses into the VMH. Statistical significance was determined by *t*-Student test. Error bars represent the SEM. * *p* < 0.05, and *** *p* < 0.001 vs. (STD or vHFD) Ad GFP. For the Western blot analysis, representative images for all proteins are shown; in the case of the loading controls a representative gel is displayed, although UCP1 protein was corrected by its own internal control (α-tubulin). In the gel images, all the bands for each picture come always from the same gel, but they may be spliced for clarity.

Next, we investigated the effect of GRP78 adenoviruses on thermogenesis. Our results showed that while GRP78 in the VMH impact neither the temperature of the BAT nor the body temperature in STD rats (Figure 2A,B), it increased BAT and body temperature in vHFD rats (Figure 2C,D). In line with this, GRP78 adenoviruses increased UCP1 protein levels in the BAT of vHFD but not STD rats (Figure 2E,F).

### 3.3. GRP78 in the VMH Stimulated the Browning of WAT of vHFD Rats

**Figure 3:**
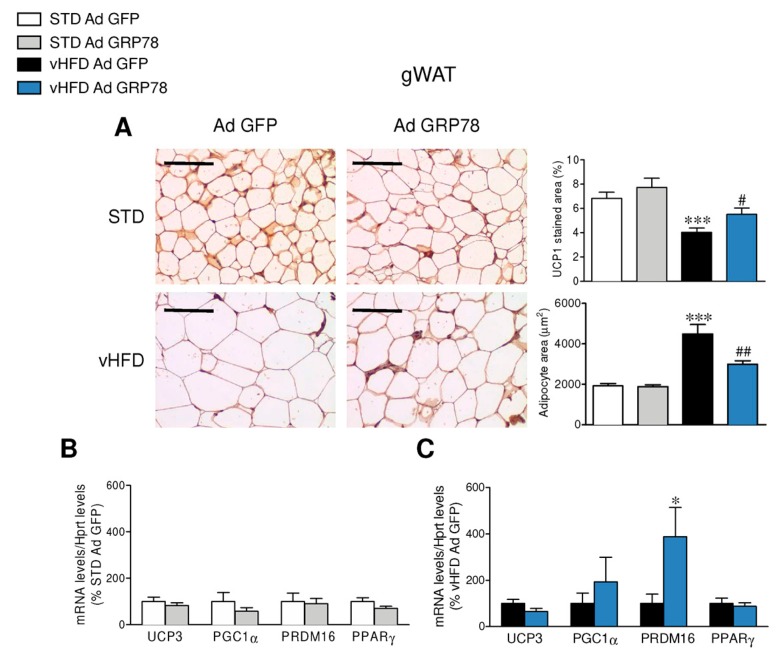
Effect of GRP78 overexpression in the VMH of vHFD rats on gonadal white adipose tissue (gWAT) browning. (**A**) Representative immunohistochemistry with anti-UCP1 antibody showing UCP1 staining (left panels; original magnification 20×; scale bar 100 µm), UCP1 stained area (right upper panels; *n* = 8–10 animals per group), adipocyte area (right lower panels; *n* = 8–10 animals per group) and (**B**,**C**) relative messenger RNA (mRNA) levels of thermogenic markers (*n* = 8–10 animals per group) in the gWAT of the STD or vHFD rats stereotaxically treated with GFP or GRP78 adenoviruses into the VMH. Statistical significance was determined by *t*-Student test or ANOVA. Error bars represent the SEM. * *p* < 0.05 and *** *p* < 0.001 vs. (STD or vHFD) Ad GFP; # *p* < 0.05 and ## *p* < 0.01 vs. vHFD Ad GFP (panel **A**).

**Figure 4:**
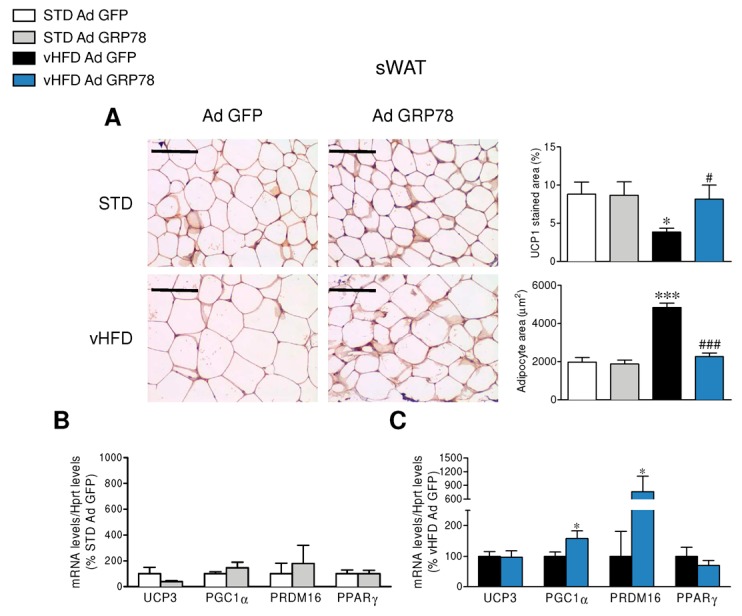
Effect of GRP78 overexpression in the VMH of vHFD rats on subcutaneous WAT (sWAT) browning. (**A**) Representative immunohistochemistry with anti-UCP1 antibody showing UCP1 staining (left panels; original magnification 20×; scale bar 100 µm), UCP1 stained area (right upper panels; *n* = 8–10 animals per group), adipocyte area (right lower panels; *n* = 8–10 animals per group) and (**B**,**C**) relative mRNA levels of thermogenic markers (*n* = 8–10 animals per group) in the sWAT of the STD or vHFD rats stereotaxically treated with GFP or GRP78 adenoviruses into the VMH. Statistical significance was determined by *t*-Student test or ANOVA. Error bars represent the SEM. * *p* < 0.05, and *** *p* < 0.001 vs. (STD or vHFD) Ad GFP; # *p* < 0.05 and ### *p* < 0.001 vs. vHFD Ad GFP (panel **A**).

To further investigate the effect of GRP78 overexpression in the VMH of vHFD rats, we studied its impact on the browning of WAT. The administration of GRP78 in the VMH of vHFD rats decreased adipocyte size and increased UCP1 immunostaining in the gWAT when compared to GFP vHFD rats (Figure 3A), which was also observed in the sWAT (Figure 4A). Comparable data were found when the same samples were analyzed by real-time PCR, which confirmed the elevation of thermogenic markers in the gWAT and sWAT of vHFD but not STD rats treated with GRP78 adenovirus within the VMH (Figure 3B,C and Figure 4B,C).

### 3.4. GRP78 in the VMH Ameliorated the Metabolic Phenotype of vHFD Rats

**Figure 5:**
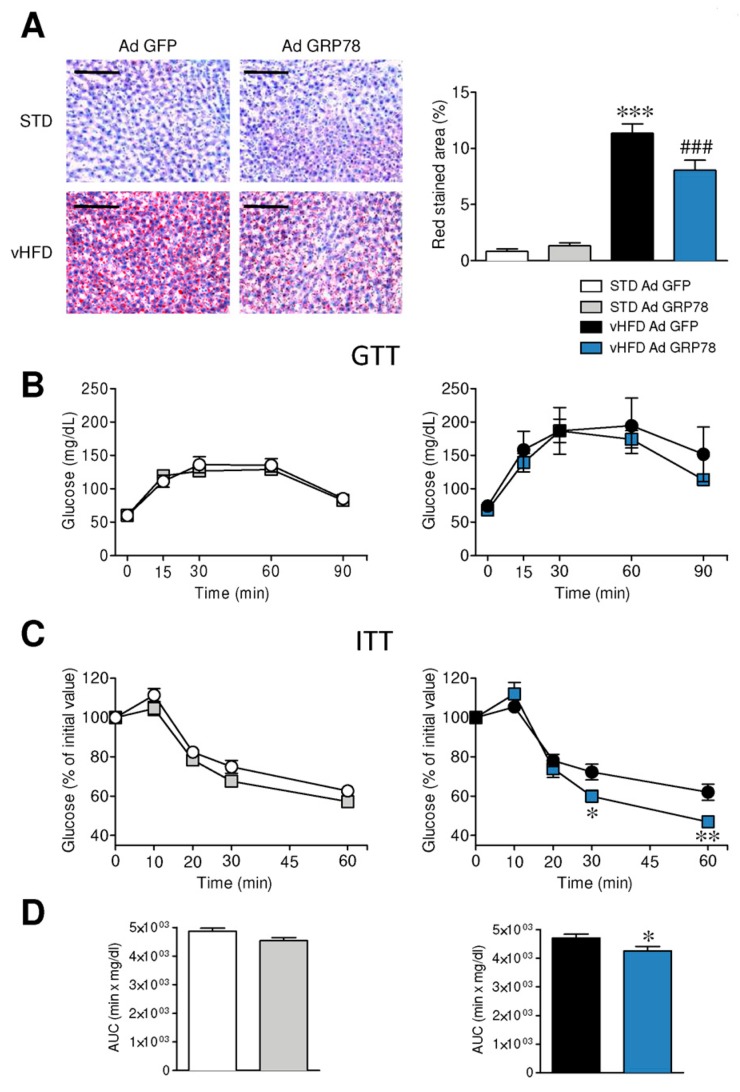
Effect of GRP78 overexpression in the VMH of vHFD rats on hepatic steatosis, glucose homeostasis and insulin sensitivity. (**A**) Representative oil red O stained liver sections (left panel; scale bar 100 µm) and their quantification (right panel; *n* = 7–10 animals per group), (**B**) glucose tolerance tests (GTT) (*n* = 8–10 animals per group), (**C**) insulin tolerance tests (ITT) (*n* = 9–11 animals per group) and (**D**) the area under the curve (AUC) (*n* = 9–11 animals per group) from ITT of the STD or vHFD rats stereotaxically treated with GFP or GRP78 adenoviruses into the VMH. Statistical significance was determined by *t*-Student test or ANOVA. Error bars represent the SEM. * *p* < 0.05, ** *p* < 0.01 and *** *p* < 0.001 vs. (STD or vHFD) Ad GFP; ### *p* < 0.001 and vs. vHFD Ad GFP (panel **A**).

Subsequently, we wanted to investigate whether the effects of GRP78 manipulation could ameliorate the metabolic comorbidities of obesity, such as hepatic steatosis and impaired glucose homeostasis. Our data showed that GRP78 adenoviruses reduced vHFD-induced hepatic lipid content (Figure 5A). We also evaluated the impact of VMH GRP78 overexpression on glucose tolerance and insulin sensitivity in STD and vHFD rats. Our data showed that administration of GRP78 into the VMH did not impact glucose tolerance in either STD or vHFD rats (Figure 5B). However, the insulin resistance that characterizes vHFD rats was ameliorated by GRP78 adenovirus in the VMH, while it did not affect STD rats (Figure 5C,D). Overall, these data indicate that the targeting of GRP78 in the VMH induces not only a feeding independent improvement of body weight, but also an amelioration of the metabolic complications associated with obesity. 

### 3.5. The Body Weight Loss Caused by VMH GRP78 Is Positively Correlated with the Degree of Obesity

**Figure 6:**
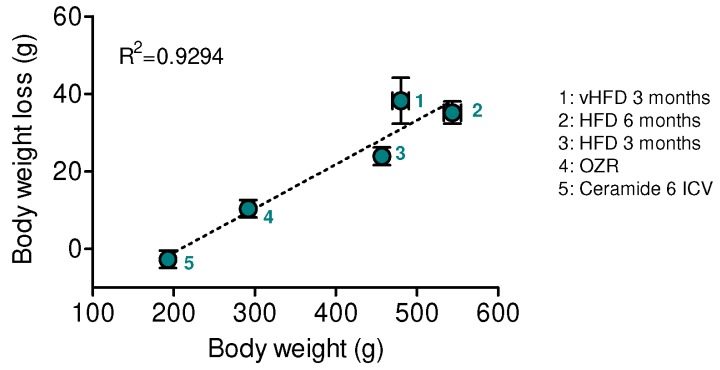
Effect of GRP78 overexpression in the VMH of different rat models of obesity. Correlation analysis (linear regression) between obesity degree and body weight loss in rats fed a vHFD (60% fat; labelled as 1 in the graph; *n* = 8 animals per group), rats fed a HFD for 6 months (45% fat; labelled as 2 in the graph; *n* = 20 animals per group) [23], rats fed a HFD for 3 months (45% fat; labelled as 3 in the graph; *n* = 27 animals per group) [23], obese Zucker rats (labelled as 4 in the graph; OZR; *n* = 37 animals per group) [17,23], and rats receiving intracerebroventricular (ICV) injections of ceramide 6 (labelled as 5 in the graph; *n* = 23 animals per group) [17], stereotaxically treated with Ad GRP78 adenoviruses into the VMH; in all the cases the adenoviral treatment lasted 7 days. Error bars represent the SEM.

Finally, we aimed to investigate whether the effect of GRP78 in the VMH on body weight could be dependent of the degree of obesity. Recent data from our group demonstrated that targeting the chaperone in this hypothalamic nucleus decreased obesity in rats centrally treated with ceramides, obese Zucker rats (OZR), and rats fed a HFD with a lower content of fat (45%) during different periods (3 and 6 months) [17,23]. Therefore, we plotted the GRP78-induced weight loss against the starting body weight of the animals when the adenoviral treatment into the VMH was given. Our data showed that there was a positive correlation between the catabolic action of VMH GRP78 and the degree of obesity, being the largest effects of GRP78 observed in the most obese models, namely long-term HFD 45% and vHFD rats (Figure 6).

## 4. Discussion

This study highlights the role of the chaperone GRP78 in the VMH, a major hypothalamic nucleus implicated in the modulation of brown fat thermogenesis and browning [3,6,7]. We show that GRP78 induces a beneficial effect on vHFD-induced obesity and on its associated metabolic complications, such as hepatic steatosis and insulin resistance.

The ER is a cellular site of newly synthesized proteins. Any alterations in ER organization or in chaperone activity lead to an accumulation of unfolded proteins, activating the unfolding protein response (UPR) [34,35,36]. Numerous studies have revealed a strong correlation between ER stress, obesity, and its associated comorbidities at peripheral level [12,32,33,37,38]. In the same line of findings, it has been exposed that obesity and overnutrition-induced inflammation could promote hypothalamic ER stress, leading to insulin and leptin resistance and, ultimately to weight gain [15,16,20,21,23,39,40]. Interestingly, improving protein folding by chemical chaperones and/or genetic manipulation of UPR helps to restore leptin and insulin signaling, subsequently normalizing the body weight [15,16,17,20,21,23,39,40]. Moreover, it is well described that the ceramide-induced lipotoxicity can engender hypothalamic ER stress, consequently leading to body weight gain, glucose intolerance, and decreased BAT thermogenesis, as direct consequence of the reduction of the sympathetic tone [17,22,24]. Notably, the central action of ceramides can be reversed by decreasing ER stress, specifically into the VMH [17,23,24].

We have recently demonstrated that the chaperone GRP78, which is located upstream of the UPR pathway [34,35,36], had beneficial effects on obesity through its direct action in the VMH. This finding was confirmed in several models: (i) rats centrally treated with ceramides [17], (ii) short-term (3 months) and long-term (6 months) diet-induced obese (DIO) rats [23], (iii) as well as in genetic models, such as in OZR [17,23]. Therefore, the objective of this study was to investigate the efficiency of this genetic manipulation in a model of morbid obesity, namely rats fed with vHFD. The current observed mechanisms correspond to the ones already described (feeding-independency associated to BAT and browning activation), leading ultimately to (i) a significant and maintained body weight loss and to (ii) a metabolic improvement, as demonstrated by decreased steatosis and increased insulin sensitivity. Moreover, we have shown that the amplitude of the effect was positively correlated to the degree of obesity. Therefore, our data strengthen the idea that the modulation of ER stress within the VMH by GRP78 is a feeding-independent central mechanism regulating BAT thermogenesis and WAT browning. All these findings suggest that the upstream central control of both processes may be a potential strategy against obesity and its associated morbidities.

The clinical relevance of these findings is intriguing. Chemical chaperones, common compounds used for the reduction of ER stress, have the potential to improve leptin resistance in overnutrition and overweight conditions. As an example, tauroursodeoxycholic acid (TUDCA) or 4-phenyl butyric acid (4-PBA), both described to decrease ER stress and enhance leptin sensitivity in vitro and in vivo [20,21,23], can reinforce weight loss and anorectic effects when co-administered with exogenous leptin [21]. Interestingly, TUDCA and 4-PBA have been approved by the U.S. Food and Drug Administration (FDA) due to their high safety profiles in humans [41,42], thus providing an emerging therapeutic approach for metabolic diseases. Recent data obtained by our group also demonstrate that TUDCA induces BAT thermogenesis and WAT browning [23]. Thus, rodent data support that the chemical chaperones might be reprofiled to treat metabolic syndrome and it is tempting to speculate that targeting hypothalamic ER stress, and more specifically GRP78, may be a suitable strategy for the treatment of obesity and associated comorbidities.

## Supplementary Materials

The following are available online at http://www.mdpi.com/2073-4425/9/7/357/s1. Table S1: Primers and probes for real-time PCR (*TaqMan^®^*) analysis.
