# Supplementary material for: Genetic Targeting of GRP78 in the VMH Improves Obesity Independently of Food Intake
Source: Genes (Basel). 2018 Jul 17;9(7):357. doi: 10.3390/genes9070357 (PMC6070933; doi:10.3390/genes9070357)
Supplement: Supplementary file 1 [file genes-09-00357-s001.zip › genes-323693-supplementary.pdf]

Supplemental Table 1. Primers *and probes for real-time PCR (TaqMan®) analysis.*

mRNA	GenBank Accession Number		Sequence
**CIDEA**	NM_001170467.1	Assay ID	Applied Biosystems TaqMan® Gene Expression Assays
			Assay ID Rn04181355_m1
**HPRT**	NM_012583	Fw Primer	5'-AGCCGACCGGTTCTGTCAT-3'
		Rv Primer	5'-GGTCATAACCTGGTTCATCATCAC -3'
		Probe	FAM-5'- CGACCCTCAGTCCCAGCGTCGTGAT 3'-TAMRA
**PGC1α**	NM_031347	Fw Primer	5'-CGATCACCATATTCCAGGTCAAG-3'
		Rv Primer	5'-CGATGTGTGCGGTGTCTGTAGT -3'
		Probe	5'-AGGTCCCCAGGCAGTAGATCCTCTTCAAGA -3'
**PPARγ**	NM_013124	Fw Primer	5'-TGGGCCAGAATGGCATCTC-3'
		Rv Primer	5'-CTGATGCACTGCCTATGAGCACTTCACA-3'
		Probe	FAM-5'-CTAACTCCCAGAAAAGCAAGCAA-3'-TAMRA
**PRDM16**	XM_008764418.1	Assay ID	Applied Biosystems TaqMan® Gene Expression Assays
			Assay ID Mm01266512_m1
**UCP3**		Assay ID	Applied Biosystems TaqMan® Gene Expression Assays
			Assay ID Rn00565874_m1
